# Unveiling the potential of fine arts education in enhancing resilience among Chinese gifted students

**DOI:** 10.3389/fpsyg.2024.1402725

**Published:** 2024-11-12

**Authors:** Zhe Dong, Ying Zhang

**Affiliations:** Institute of Art & Design, Shandong Women's University, Jinan, China

**Keywords:** fine arts education, resiliency, gifted student, creativity, self-efficacy, Chinese university

## Abstract

**Introduction:**

The current study aimed to explore the complex dynamics influencing the resilience of gifted Chinese students, emphasizing the interconnected roles of fine arts education (FAE), creativity, and self-efficacy (SE). By examining the transformative potential of FAE, this research highlighted creativity and SE as key mediating factors for resilience among Chinese gifted students.

**Methods:**

This study involves administering a structured set of questions to a sample of gifted young adults, thus achieving a high response rate of 93.21%. The Maus scale (1938) has been used to measure FAE, the Connor-Davidson Resilience scale assesses resilience, while creativity and SE are measured using the Kaufman scale and Schwarzer and Jerusalem scale, respectively. Statistical analyses includes a correlation matrix, ANOVA, confirmatory latent factor model tests, and mediation effect analysis for resilience and creativity. Data analysis has been conducted by using the SPSS Statistics 29.0, bootstrap, and AMOS 25.0 software.

**Results:**

Current findings determines a significant role of FAE in enhancing emotional resilience, creativity, and SE among gifted students. This study also finds the importance of FAE in building essential coping mechanisms to address intellectual and emotional challenges specific to this group.

**Discussion:**

This study proposes to integrate FAE into educational curricula to better support the intellectual and emotional needs of gifted young adults, thereby facilitating the development of coping skills crucial for their emotional wellbeing. Additionally, this study indicates the importance of art therapy and global initiatives in boosting the resilience, while preparing gifted students to face future challenges with enhanced psychological resilience.

## 1 Introduction

Giftedness provides a double-edged sword for young individuals and is a hallmark of exceptional intellectual abilities. This characteristic propels young minds toward excellence, while subjecting them to elevated levels of stress and scrutiny (Mhlolo and Ntoatsabone, 2023). In the realm of education, the journey of gifted students often evolves with a blend of distinctive challenges and unparalleled opportunities. This scenario is also found in other sectors, higher margin requirements improve overall returns and reduce volatility (Subrahmanyam et al., 2024). This scenario reflected the challenges of gifted students in Germany, whereas this emphasized the need for social support and resilience to combat isolation. Also, Choi et al. (2023) elaborately discussed the mediating effects of academic resilience of gifted youth in relation between academic burnout and school adaptation.

### 1.1 Foundational aspects

Particular to the context of China, where academic standards are notably high, gifted pupils sometimes find themselves traversing through a landscape of rigorous expectations and intense academic pressures. Like, Zhu Ying, a gifted student at Peking University, China, faced significant challenges despite her academic prowess. Dr. Wei Liu's study on high-achieving Chinese students documented her struggles with academic pressure and social isolation. This case proved the need for resilience training and support systems to address these effectively in Chinese academic institutions (Chen et al., 2024). However, within this crucible of academic rigor, there is a profound potential for transformation that extends well beyond the conventional boundaries of academia (Frazier et al., 2021). In this regard, several well-established articles have shed light on this transformative potential by considering the intricate interplay between fine arts education (FAE), creativity, and self-efficacy (SE), thus recognizing their collective role to frame resilience among Chinese gifted pupils (Alabbasi et al., 2023).

SE refers to the belief in one's ability to successfully complete tasks and achieve goals. Many researchers show that individuals with higher SE face challenges with greater confidence, while those with lower SE tend to avoid obstacles (McKay et al., 2020). Also, they describe SE to be a crucial component of psychological resilience, influencing how individuals manage academic, professional, and personal aspects of their lives (Schunk, 2023). On the other hand, creativity in students refers to their ability to generate original ideas, solve problems with innovation, and think critically. This aspect develops their adaptability, enhances learning, and promotes self-expression. Thus, researchers find creativity to be vital for encouraging independent thinking and preparing students for diverse real-world challenges (Morse et al., 2021).

Researchers also describe that gifted children face distinctive challenges that affect their overall development (Oertwig et al., 2019). Perfectionism often leads to stress and anxiety as they set unrealistically high standards for themselves, whereas social isolation can occur because their advanced abilities can make this difficult to connect with peers, thus impacting their social and emotional growth (Xu W. et al., 2024). Additionally, underachievement results from a lack of challenging material or a disconnect between their interests and the curriculum. A high-intensity withdrawal-motivation emotion sometimes lead to duration overestimation in select temporal tasks, thus seeking attention's role in time perception (Zhang L. et al., 2024). High emotional intensity is common, which often leads to increased stress in managing emotions (Gao et al., 2024). Moreover, a lack of support from educators untrained in addressing their needs can lead to unmet academic and emotional requirements (Huang et al., 2020). This way, the present study determines that developing resilience is essential for overcoming these challenges for the gifted young adults. This variable enables them to cope with failure, manage stress, and build emotional strength, thus enhancing social skills and maintaining motivation to ultimately support their real-life success (Chen et al., 2023).

More precisely, one needs to determine the intricate mechanisms inherent to build and improve the resilience of gifted students (Halberstadt et al., 2020), while several articles find the pivotal role of FAE as a catalyst for the said development of young adults (Huang et al., 2020). Thus, this is essential to explore the interplay among FAE, creativity, and SE. Also, one should measure the impacts of artistic pursuits in enhancing the academic performance of gifted students (Reed et al., 2020). Thus, this study finds very few existing articles that had considered the multifaceted impacts of FAE on the resilience of gifted students. Also, exploitation of information related practices in public surveillance can enhance the detection and response understanding (Zhang D. et al., 2024). Because FAE is often perceived as a leisure activity, this is essential to underscore its profound transformative power by transcending mere recreation to become a cornerstone of resilience-building among young individuals (Li et al., 2023). Through empirical investigation, this study plans to shed light on how FAE empowers Chinese gifted students to confront academic pressures, discover their identities, and cultivate creative problem-solving skills essential for both the academic and real-life success.

### 1.2 Research questionnaire

Aforementioned aspects lead the present study to determine some research questions as follows:

- How does participation in FAE influence the emotional resilience and SE of gifted young adults, and what role does creativity have in moderating and/or directly affecting this relationship?
- To what extent does creativity moderate the relationship between FAE and emotional resilience in gifted young adults, and how does emotional resilience impact the relationship between SE and FAE?
- What is the effect of art-based emotional support programs by higher educational institutions on the SE and stress management of gifted young adults?
- How do extracurricular artistic activities and community-based art programs enhance the social adaptability and creative problem-solving skills of gifted young adults?

This way, the present study finds that creativity is a construct that involves the development of novel, innovative, and insightful solutions (Morse et al., 2021), whereas one should examine the moderating influence of SE by focusing on its amplification of the positive effects of FAE on resilience. Therefore, this study aims to explore how participation in FAE influences the emotional resilience and SE of gifted young adults, with a focus on creativity as both a moderating and direct factor. In contrast to existing articles, this study plans to examine how art-based emotional support programs and extracurricular artistic activities aid stress management, social adaptability, and creative problem-solving in gifted students at institutions of higher education. Current findings shall guide educational institutions, policymakers, and other stakeholders in developing policies that promote the wellbeing of gifted students. Unlike previous research, which has examined these factors individually, this study will comprehensively investigate their interconnections and collective impact on resilience within the Chinese educational context. Also, by identifying current challenges and future opportunities, this study seeks to provide practical insights that inform educational practices to better support the real-life wellbeing of gifted individuals.

### 1.3 Arrangement of the paper

Rest of the present paper is arranged as follows. Section 2 provides a concise overview of different research aspects gleaned from existing literature. Whereas this scenario sets the stage for current research, Section 3 goes deep into the key areas of this study, and thus formulates four major hypotheses on basis of the interplay in improving resilience among gifted young adults. In Section 4, this study discusses the research methodology by outlining the approach taken to address the said research questions and validate the proposed hypotheses. This study performs several statistical computations and provides the corresponding results in Section 5. Next, Section 6 tests the validation of the proposed hypotheses on basis of those results. Later, this study extracts a number of useful acumen for various stakeholders along with their global implications. Finally, Section 7 makes the conclusions of this study while identifying its limitations and potential future research scopes.

## 2 Literature review

The present section provides a brief review of current literature in some major aspects as follows.

### 2.1 FAE of young individuals

Education in any form has a pivotal role in individual development. Within this realm, various forms of FAE, such as sculpture, dance, decorative art, digital art, cinema, and several others, emerge as a crucial building block for the progress of societies (Jin and Ye, 2022). Thus, FAE serves as a potent route to express the identity, culture, emotion, lifestyle, and societal experience of human beings within the educational sector. Historically, Ryff and Keyes (1995) developed the framework for the psychological wellbeing while identifying six interconnected, distinct aspects in the self-fulfillment and self-improvement realm, namely personal growth, self-acceptance, positive relationships, autonomy, environmental mastery, and life-meaning. Later, Rand and Cheavens (2009) and many other researchers investigated the impacts of FAE on those diverse aspects.

A study on meritorious students by Morse et al. (2021) showed that to engage with various forms of arts in health research would encompass performing arts, visual arts, design, craft, literature, and cultural activities. They recognized these aspects because of their health-promoting attributes. Wherein FA related activities were found to be complex with various interventions, they joined various artistic forms associated with health benefits. Al-Hroub (2022) recommended that FAE in form of dance, music, and theater would extend numerous advantages to gifted students by developing creative and innovative thinking in themselves. Also, they found that gifted students' participation in FAE activities could improve their various skills. The study of Xu W. et al. (2024) indicated that FAE enhanced gifted students' academic performance and engagement in other activities. They showed the role of those activities in improving the overall social involvement of gifted students. Around this time, Jin and Ye (2022) advocated for the higher educational institutions to employ FAE as a medium. Their recommended approach would assist students in various life-skills, including morals, values, innovation, and aesthetics. Moreover, the merging of art education with visual culture in higher educational institutions could prepare young adults for active participation in a democratic society.

Very recently, a novel article of Xu T. et al. (2024) investigated into the impact of FA activities over undergraduate students' psychological wellbeing and showed the moderating aspects of SE and creativity. Their findings indicated that creativity and SE significantly moderated between FA activities and psychological wellbeing in China. They suggested incorporating art courses into educational curricula at various levels to enhance the creativity and SE of young adults in China. In a study focusing on engineering skills, Sharma and Kumar (2023) recommended a structured methodology to enable and equip students with confidence with FAE, like drawing, sketching, and/or painting. They demonstrated the significant influences of pre-training students in FAE on various courses, like engineering drawing and strength of materials. Thus, they emphasized a blend of concepts, including conceptual, theoretical, spatial visualization and problem-oriented concepts. Yang and Xiangming (2024) addressed the problem of insufficient emphasis on art courses in helping students develop modern skills. They found universities to be responsible for equipping scholars with these soft skills, yet many overlooked collaboration opportunities. They also utilized a qualitative single case study methodology on constructivist learning theory and design thinking and thus analyzed the impact of art courses on non-art majors' modern skill development. Although visual arts pedagogies were central to early childhood education programs, Denee et al. (2024) found that teacher SE would directly impact the delivery of visual arts curricula. They discussed a qualitative re-analysis of three Ph.D. studies, thus revealing the powerful influence of SE on teaching practice. Also, they could identify the factors and conditions that enhanced visual arts SE.

### 2.2 Resiliency of young adults

Resilience in young individuals reflects their capacity to bounce back from challenges, adversity, and trauma. This is a critical aspect of their development and wellbeing. Recent literature has put major attention on the multi-dimensional construct of resilience in the academic, social, and personal lives of young adults (Liu et al., 2024).

Li et al. (2023) conducted a cross-sectional study involving undergraduate pupils of Shandong province in China. They explored the interconnection of students' resilience with their coping styles. Their female students in medical courses were more likely to adopt positive coping styles compared to non-medical male students. The results also showed the importance of psychological education and health promotion programs aimed at enhancing psychological resilience among undergraduate students. Accordingly, they recommended a method to promote positive coping styles and overall psychological wellbeing. Around this time, Price (2022) created charts outlining various applications of resilience and wellbeing in higher education. They identified some core elements in the practice of resilience including cognitive flexibility and reflection. Their aforementioned aspects could offer some preliminary opportunities to frame the resilience-related training for the designers. In a unique study, Labrague (2021) investigated effects of pandemic-related stress on nursing students' wellbeing and life satisfaction, as mediated by resilience. Their primary data based survey of 301 nursing students in the Philippines revealed that pandemic-associated higher stress had correlated with less life satisfaction and poorer psychological wellbeing. Resilience could help mitigate the negative effects of pandemic-induced stress on nursing students' wellbeing and their life-satisfaction.

In a recent study, Liu et al. (2023) investigated the efficacy of interventions related to mindfulness in school settings using a cluster randomized controlled trial involving 92 middle school pupils. The research showcased a notable rise in psychological resilience and trait mindfulness, coupled with a reduction of psychological stress among participants for experimental group. They found trait mindfulness to be positively associated with school students' resilience, whereas both were related negatively to psychological stress. Around same time, Chua et al. (2023) conducted a comprehensive search of 11 electronic education and health-science databases. They performed a subgroup analysis and thus revealed significant effects of resilience measures on resilience prevalence. They observed higher pervasiveness for less resiliency of European nursing and dance students compared to their counterparts. Besides, Shi et al. (2023) investigated the issue of college students' smartphone addiction as an urgent problem. They empirically tested their model of teacher-student connections and addiction for smartphones. Thus, they found that the teacher-student relationships negatively predicted smartphone addiction. Their orientation could show the multifaceted nature of diverse factors influencing smartphone addiction of college students. During this period, Rivera-Romero et al. (2024) found that adolescence was a stage marked by significant changes, which was also crucial for shaping individuals' life courses. They described that in Latin America, adolescents and young adults in countries like Colombia would face unequal access to socioeconomic resources, education, and the job market. These scenarios could lead to social disadvantages and vulnerability. They found that social vulnerability and psycho-social resilience coexisted and that social support networks and community art processes could enhance resilience.

### 2.3 Self-efficacy of young individuals

Researchers refer the SE as the confidence young individuals have in their ability to effectively achieve a specific goal or bring about a desired outcome. Since long, SE has been a classical topic of discussion, whereas this belief can often be related to a specific activity (Martin, 2023).

Viewing SE beyond a demand-control goal, Chen et al. (2024) described capacity of aligning the major aims with one's potential under particular situations by playing a key role in different psychological issues. They associated the low SE with behavioral aspects of university students. The study involving first generation students of digital natives (i.e., Gen Z) by Mihelič et al. (2022) investigated smartphone use for non-academic purposes. They found that subjective norms were negatively associated with cyberloafing in class, whereas moral disengagement had a positive correlation with this. In a novel research, Kausar and Ahmad (2021) explored the correlation between psychological wellbeing, SE, and stress among the Pakistani students of performing arts. Their results indicated that female Pakistani students showed more stress in comparison to male counterparts. The practical implications of their research extended to counselors and therapists, while generating vital acumen into stress and psychological wellbeing of Pakistani adults.

Of late, Yin et al. (2023) examined the longitudinal dynamics between kindergarten teachers' SE, wellbeing, and commitment to children. Their research revealed that teachers' wellbeing positively bolstered their commitment to children over time, with SE acting as a mediating factor. They recommended incorporating positive psychology principles into social cognitive theory and thus stressed the need for longitudinal research to better understand the predictive role of SE. Longitudinally exploring relationships among academic SE, related stress, and psychological distress in Norwegian adolescents, Kristensen et al. (2023) found that academic stress could directly influence the psychological distress, whereas academic SE would partly mediate these effects. Besides, they observed some gender differences with boys experiencing stronger interpersonal effects and girls exhibiting stronger individual impacts. In this period, Jiang et al. (2024) conducted one study on the promotion of self-care behavior among adults, who were suffering from type 2 diabetes. They applied a three-arm cluster randomized controlled trial in their study. They found that self-care behavior were highly associated with participants' SE. HadaviBavili and İlçioğlu (2024) considered anatomy, which had been a crucial subject in health science. They found that passive learning methods were traditionally used in anatomy. Their article with 181 students compared existing methods and thus found both improved SE in anatomy (*p* < 0.005) together with no significant difference between methods (*p*>0.005). They identified that FAE had effectively enhanced SE and thus improved individuals' real-life skills. Around this time, Abadie et al. (2020) found that teachers prioritized mastery experiences and verbal persuasion over vicarious experiences and psychological skills, whereas significant differences in the use of verbal persuasion between studio and classroom teachers had reflected their distinct environments. They suggested to focus on the less-utilized SE sources for improving performance.

### 2.4 Creativity of young individuals

Continuing development of technology and science motivates young people to focus on creativity while acquiring various novel skills to keep up with those changes. This is well-regarded that the investment theory of creativity posits this to be influenced by diverse factors, including intellectual abilities, sufficient knowledge, creative thinking skills, personality traits, intrinsic motivation, and a conducive environment (Sternberg and Lubart, 1999).

In a novel study, Li et al. (2021) synthesized an article focusing on the features of educational environments conducive to developing creative processes. Their work showed several key themes, such as the integration of creative process skills, the significance of adaptive environments, the cultivation of a reflective culture in classrooms, and challenges related to implementation. Around the same period, Kancan et al. (2023) underscored the comprehensive development of personality as a crucial task in the modern psychology system within higher educational institutions. Their model, while being deemed as universal, allowed the addition of separate sub-levels and sub-tasks to address specific needs of educational institutions. Moreover, they made some notable contributions toward forming a universal algorithm with poly-levels for detailing goals. Focusing on nurturing mathematics related creativity and problem posing in gifted students encountering challenges, Ayvaz and Durmus (2021) demonstrated the significant efficacy of their proposed action plan in enhancing these skills. Their intervention resulted in markedly improved post-test scores in both domains, underscoring its effectiveness. Concurrently, Bahcelerli and Altınay (2023) investigated the wellbeing of students, who were enrolled in two gifted programs featuring distinct service delivery models. Their findings uncovered distinct patterns of psychological wellbeing between students in the two programs and those in the control group lacking gifted services. This sheds light on unique social phenomena and offers valuable insights into gifted students' psychological wellbeing across various educational settings.

In a recent article, Olamafar et al. (2023) explored the intersections of creativity, general intelligence, plus wisdom among gifted adolescent Iranian pupils. Their findings unveiled a noteworthy positive correlation between creativity and wisdom, showing the pivotal role of creativity in nurturing wisdom and promoting a fulfilling life. However, there was hardly any significant correlation between general intelligence and creativity in their data. Examining the predictors of inter-rater disagreement in a large dataset of pupil's creativity assessment responses, Dumas et al. (2023) constructed a model by considering 387 elementary school students and 10, 449 individual item responses. They showed that some responses, that were characterized by lower originality, greater elaboration, used task, originating from younger children or male students, were more challenging to reliably rate. They also demonstrated that, given the reliance on human judgments for assessing creativity, understanding the nuances of these evaluations was crucial. Drawing on multiple longitudinal samples of gifted students for talented education in the United States, Wai et al. (2024) underscored the importance of replication, especially of the constructive replications. They also emphasized methodological design features, particularly focusing on predictors and outcomes, while considering various aspects, such as magnitude and breadth, to comprehend the dynamics of gifted education for talented learners.

### 2.5 Critical connections of reviewed literature with current research

The recent and established literature could establish the complicated and versatile nature of creativity and its significant impact on young adults. Angela and Caterina (2020) and Li et al. (2021) showed the importance of nurturing creativity and creating conducive educational environments, which confirmed the moderating role of creativity in the relationship between FAE and resilience. Olamafar et al. (2023) illustrated a positive correlation between creativity and wisdom, thus supporting the direct role of creativity in the relationship between FAE and SE. Articles of Ayvaz and Durmus (2021) and Bahcelerli and Altınay (2023) also demonstrated how creativity impacted academic and psychological outcomes, thereby reinforcing that artistic endeavors enhanced resiliency, which in turn would affect the relationship between SE and FAE.

Building on current review, the present study found that FAE significantly contributed to personal and academic development. Specifically, Morse et al. (2021) and Al-Hroub (2022) emphasized the role of creativity in educational settings, while advocating for its moderating effect on FAE's impact on resilience. Moreover, Xu T. et al. (2024) and Xu W. et al. (2024) provided evidence that both resiliency and creativity influenced SE and academic performance among gifted students. These connections showed the importance of integrating FAE into educational practices to enhance both emotional and cognitive development for gifted young adults.

Furthermore, recent literature discussed the critical role of resilience in young adults, particularly in academic and personal contexts. Taylor et al. (2020) and Li et al. (2023) illustrated how resilience helped them in coping with stress and adversity, thus establishing its direct impact on the relationship between SE and FAE. Besides, Liu et al. (2023) and Chua et al. (2023) showed the benefits of mindfulness and psychological resilience, which could affirm that engaging in artistic endeavors enhanced resiliency. In this regard, Labrague (2021) and Shi et al. (2023) supported the relevance of resilience in mitigating stress, which provided a foundation for understanding the role of creativity in this dynamic. All these developments together with the research questions call this study for framing the hypotheses as below.

## 3 Proposed hypotheses

### 3.1 Fine arts education and resiliency of gifted students

Researchers find some significant connections between FAE and overall wellbeing of those students (Reed et al., 2020). Particularly, engaging in various forms of FAE is a powerful mean for nurturing resiliency among students (McKay et al., 2020). Researchers show the profound impacts of those engagements on the multifaceted development of resiliency among young adults. The said connections can provide a distinctive platform for emotional expression and exploration of gifted students. Through participation in sculpture, ceramics, painting, and several others, gifted students can embark on a journey of self-discovery and emotional expression. This process develop their emotional intelligence and allows them to gain a deeper understanding of their feelings. When those students are proficient in navigating their emotional landscapes through FAE, the resiliency within them extends beyond academic challenges to encompass various facets of their lives (Price, 2022).

The immersive nature of FA activities serves as a refuge for gifted students. Whereas they acquire effective stress coping mechanisms with FA activities, these activities provide them with a sanctuary from the pressures of academic expectations (Li et al., 2023). FA engagement is thus a therapeutic outlet and allows gifted students to channelize the stress into creative expressions. All these aspects can significantly contribute to develop a resilient mindset in the face of adversity for young adults. Additionally, engaging actively in Fine Arts enables students to take risks, express their unique perspectives, and display their talents (Pavia et al., 2021). This empowerment can additionally contribute to the development of SE and confidence. For young pupils grappling with high expectations and self-doubt, FAE related pursuits act as a mean to build stronger sense of self-worth. This heightened confidence turns to be a foundational element of resilience, thereby empowering them to confront challenges both within and beyond the academic realm (Ermis and Imamoglu, 2019).

Particularly, the impacts of FAE can be far-reaching on the resiliency for gifted students. Typically, FAE offers a meaningful context for navigating questions of identity and finding purpose in their academic and personal journeys (Ermis and Imamoglu, 2019). Beyond developing emotional expression, stress coping, and confidence building, these activities also help cultivate problem-solving skills. Thus, FAE becomes a crucial transformative element in students' developmental journeys. Aforementioned intricate relationships motivate the present study to propose the following hypothesis:

**H1:** Engaging in artistic endeavors enhances the resiliency within gifted students.

### 3.2 Mediating role of creativity between gifted students' fine arts education and resiliency

Creativity that researchers describe as a diagnosable and restructurable process involving various analytical procedures, is linchpin to decipher the complex dynamics between gifted students' participation in any form of FAE and their capacity to rebound from different real-life based challenges. Numerous researchers have found that pupils' involvement in diverse creative endeavors would enhance their quality of education and life-decisions (Reed et al., 2020). Creativity is thus an integral part to restructure the cognitive processes and thus develop adaptability for young adults.

Typically, the journey of gifted individuals from adolescence to adulthood involves navigating challenges, such as higher education, job hunting, relationship shifts, and long-term aspirations. These challenges make students' growing phase to be stressful with unique stressors and increased vulnerability to challenges (Huang et al., 2020). Effective coping is associated with greater resilience, whereas difficulties in managing this transition can result in psychological consequences. Recognizing these costs emphasizes the urgent need to cultivate resilience in young adults for their wellbeing and success. In this regard, creativity plays a vital role in building resilience among gifted students (Park and Cha, 2023). Creativity improves their problem-solving skills, offers an expressive outlet for emotions, and improves collaboration. Consequently, the engagements in creative activities, particularly through FAE, equip gifted pupils with some invaluable tools to navigate challenges and contribute to both academic excellence and emotional wellbeing (Xu T. et al., 2024).

Moreover, convergence of gifted students' creativity, diverse FA related engagements, and resilience in overcoming challenges forms a captivating field within the domains of education and psychological development. Because gifted students possess unique cognitive abilities and creative potentials, they need to traverse an educational trajectory requiring a nuanced examination of factors that will improve their resilience (Sappa and Barabasch, 2020). The said triadic relationship can have immense practical implications for educators, psychologists, and policymakers aiming to enhance the wellbeing and success of the gifted pupils. Aforementioned considerations lead the present study to frame the following hypothesis:

**H2:** Creativity acts as a moderating factor in the relationship between gifted students' participation in FAE and their resiliency.

### 3.3 Moderating role of resiliency between self-efficacy and fine arts education of gifted students

Current study plans to determine the interaction between FAE, SE, and the mediating role of resiliency among gifted students. FAE serves as a cornerstone for gifted pupils to refine their creative talents across disciplines, such as visual arts, music, and theater. Through structured curriculum and hands-on experiences, FAE provides a platform for gifted students to cultivate their artistic skills, express creativity, and pursue their passion for arts. Concurrently, SE emerges as a critical element of gifted students' artistic journey, while reflecting their confidence in their ability to excel in artistic endeavors (McKay et al., 2020). Researchers find that the extent to which FAE influences SE, is mediated by the level of resiliency of those pupils.

Here, resiliency is referred as the capacity to adapt positively and rebound from adversity. This plays a pivotal role in shaping gifted students' responses to various challenges to be encountered during artistic pursuits (Rivera-Romero et al., 2024). When faced with setbacks such as criticism, failure, or competition, resilient pupils demonstrate the ability to persevere, maintain a positive outlook, and continue striving toward their goals (Liu et al., 2023). Thus, in the context of FAE, resiliency influences how gifted students interpret and navigate the challenges inherent in their artistic development. High levels of resiliency enable them to view setbacks as opportunities for growth, whereas lower resiliency hinders gifted students' ability to cope with life-challenges.

Understanding the mediating role of resiliency sheds light on the complex relationship between SE and FAE. By developing resilience in gifted individuals, educators can empower students to overcome obstacles, capitalize on learning opportunities, and cultivate robust SE beliefs that support their long-term success in the FA domain (Labrague, 2021). Strategies to enhance resilience often include creating supportive learning environments, promoting a growth mindset, teaching coping skills, and encouraging reflection on setbacks (Reed et al., 2020). Through these efforts, educators can equip gifted students with the tools and mindset necessary to navigate the challenges of artistic pursuits, develop their creative potential, and nurture their confidence in their artistic abilities. This observation leads the present study to frame the following hypothesis:

**H3:** Between gifted students' SE and FAE, resiliency plays the role of a mediator.

### 3.4 Moderating role of creativity between FAE and SE of gifted students

The interplay between FAE, creativity, and SE of gifted students represents a multifaceted and dynamic relationship within the educational context. FAE serves as a rich platform for nurturing the diverse talents and interests of gifted individuals across various artistic domains, including visual arts, music, theater, and dance. This provides them with a structured yet flexible environment to explore, experiment, and refine their artistic skills and expressions. Here, the main aspect is SE, which researchers refer as a psychological construct rooted in Albert Bandura's social cognitive theory (Angela and Caterina, 2020). For gifted students, SE in the realm of fine arts plays a crucial role in shaping their artistic development, creative confidence, and overall sense of accomplishment. However, the impact of FAE on SE is hardly uniform across all gifted pupils.

Researchers find that creativity of an individual varies widely among gifted students and significantly shapes their experiences within FAE. For highly creative individuals, FAE can serve as a potent catalyst for bolstering SE (Xu T. et al., 2024). These students frequently have an inherent affinity for artistic exploration, innovation, and experimentation. FAE provides them a stimulating environment, where they can refine their skills, move deeply into artistic concepts, and perfect complex techniques (Ayvaz and Durmus, 2021). Their innate creativity fuels their passion for artistic expression, propelling them to push boundaries, embrace challenges, and persevere in the face of setbacks, thereby enhancing their SE beliefs.

On the other hand, for gifted students with lower levels of creativity, the impact of FAE on SE is sometimes more nuanced and variable. These individuals face greater challenges in engaging with the material, conceptualizing original ideas, or expressing themselves artistically (Olamafar et al., 2023). In all these scenarios, personalized support, differentiated instruction, and targeted interventions are essential to scaffold their learning, cultivate their creative potential, and bolster SE. By customizing teaching methods, providing opportunities for skill development and creative exploration, and developing a supportive learning environment, educators can help less creatively inclined gifted students build greater confidence in their artistic abilities and thus enhance their sense of SE.

In essence, the moderating role of creativity underscores the need for a nuanced and individualized approach to FAE for gifted students. By recognizing and leveraging the diverse strengths, interests, and learning styles of each student, educators and practitioners can create inclusive and empowering learning experiences that develop creativity, nurture SE, and cultivate lifelong appreciation for the arts. All these compel the present study to consider the following hypothesis for Chinese gifted students:

**H4:** The relationship between FAE and SE among gifted students is mediated by their level of creativity.

**Figure 1:**
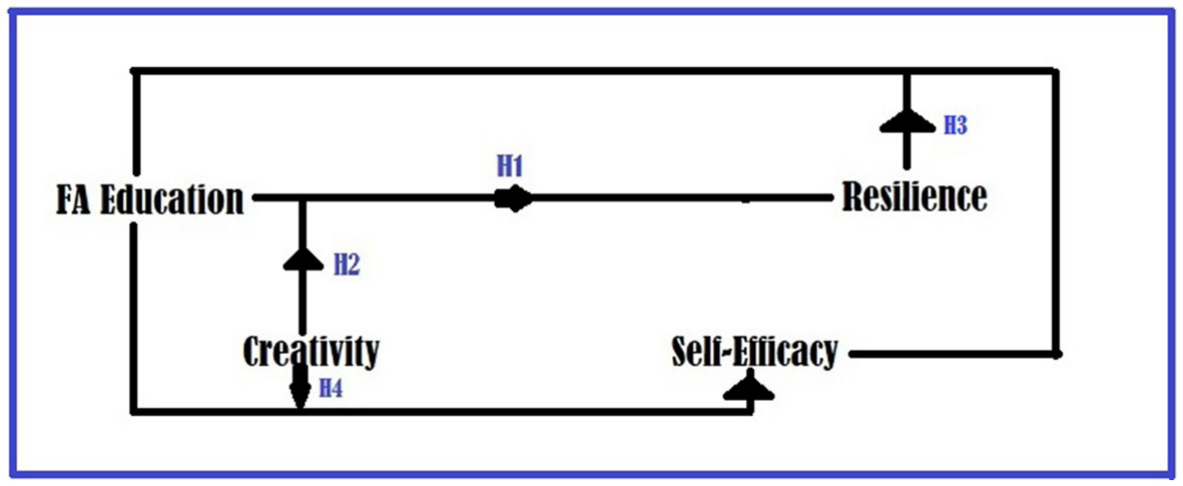
Interconnecting the hypotheses of this study.

In this regard, one can maneuver Figure 1 for the interconnections among the proposed hypotheses.

## 4 Research methodology

The present study conducts the data analysis in some established software, including *SPSSStatistics* 29.0 and *Bootstrap*. Additionally, this study utilizes the *AMOS* 25.0 software for the implementation of confirmatory factor analysis (CFA) and other analysis.

### 4.1 Description of participants

Between May 2023 and July 2023, this study conducted a primary data-based research survey involving gifted students from eleven Chinese universities, spanning both public and private institutions. The focus was on students enrolled in various FA courses. The survey was hosted on Google Docs, and the link was distributed via email and several social media platforms including the platforms of WeChat, Douban, Weibo, and Zhihu.

To determine which students were considered *gifted*, this study employed the Chinese concept of *tiancai*, which should mean exceptional talent bestowed by heaven. In practical terms, Chinese people would assess giftedness on basis of the participants' self-reported performance on the Gaokao, which was the rigorous Chinese college entrance examination. This examination would typically be considered as a key indicator of academic prowess in China, whereas this study took the median Gaokao score threshold of 645 to identify the gifted students of those institutions. This study could determine the said threshold based on the distribution of scores in those eleven Chinese universities. By choosing this specific cutoff, this study could ensure that students with exceptional academic performance only, as reflected by their Gaokao scores, would be included in the gifted category.

This approach effectively preserved a clear distinction between high-achieving students and their peers, thereby mitigating the risk of conflating responses from less qualified participants. Additionally, using this threshold could provide a robust and culturally relevant measure of giftedness, while aligning with both academic and cultural standards of excellence in the Chinese context. Employing a standardized and nationally recognized measure such as the Gaokao ensured a consistent benchmark for evaluating academic excellence across various institutions and regions. This method also minimized the influence of subjective interpretations of academic performance and/or institutional prestige, thereby enhancing the reliability of current findings.

Moreover, right from the start of the survey, this study informed participants about the details of their involvement and adopted anonymity policy before they began the survey, thereby addressing the ethical considerations. This study provided clear information about withdrawal procedures, while allowing participants to exit the survey and/or study at any time without any type of penalty. Besides, at the end of the questionnaire, participants received feedback and debriefing, which clarified the research objectives and reinforced the confidentiality of their responses. This approach could address respondents' ethical concerns by providing a clear understanding of their role, while ensuring transparency along with the opportunity to ask questions and/or raise concerns about their participation. By the end of July 2023, this study could gather data of 545 gifted students, among which 37 responses were deemed inadequate. This resulted in 508 viable answers with a 93.21% questionnaire recovery rate.

### 4.2 Measurement of data

Researchers describe that FAE can cover a wide range of formats and/or methodologies, such as painting, sculpture, drawing, printmaking, photography, ceramics, and art history. Participants receive clear instructions on how to complete each section, whereas each of these aspects provides some unique opportunities for artistic exploration, skill development, cultural enrichment, and personal growth. In this regard, this study utilized the 19−item Maus scale (Maus, 1938) to measure the independent variable FAE, which was originally designed to capture diverse artistic formats such as painting, sculpture, and art history, and thus assess various aspects of FAE in early 20th century. Current questionnaire rated each item on a five-point Likert scale, ranging from 1 (i.e., strongly disagree) to 5 (i.e., strongly agree).

However, given the significant evolution of FAE and the cultural differences between the original Maus scale of 1938*s* and the present-day Chinese university context, this was essential to adapt the scale methodically to include traditional Chinese art forms, including painting, calligraphy, and others. The content validity was ensured through expert consultation, whereas this study initially conducted a pilot test with a small group of Chinese FA students to refine the instrument, which led to enhance clarity and relevance. The feedback from this pilot test was used to make necessary adjustments, ensuring that the final instrument was well-suited for measuring FAE participation among Chinese university students. This careful adaptation process ensured that the said scale would maintain its foundational integrity while being reliable for measuring FAE in the context of modern Chinese academia.

To assesses various dimensions of resilience, including adaptability, perseverance, and personal control of respondents, researchers have employed the well-established Connor-Davidson Resilience scale (CD-RISC) scale, comprising 25 items, and thus evaluated the resiliency of young adults on a 5−point scale ranging from 0 (i.e., never) to 4 (i.e., always), with higher scores reflecting greater resilience (Connor and Davidson, 2003). The CD-RISC had demonstrated significant adaptability across diverse populations and cultural contexts. For example, the study by Campbell-Sills and Stein (2007) showed its effectiveness in capturing resilience traits in various settings for college students. Ran et al. (2020) examined the role of resilience in academic achievement among high school students in China. Thus, the CD-RISC scale had been highly reliable and validated across diverse cultural contexts, thus making this an effective tool for accurately measuring resilience in Chinese gifted students, whereas the strong reliability, convergent, discriminant, and concurrent validity support its use in educational research. Next, this study assessed the moderating variable, creativity, through a 50−item scale developed by Kaufman (2012), while adopting the 50− item scale of Schwarzer and Jerusalem (1995) for measuring the SE of Chinese gifted students. Responses are given on a five-point Likert scale, with options ranging from 1 (i.e., not at all true) to 5 (i.e., very true). Notably, Kaufman (2012) scale should evaluate various dimensions of creative thinking and problem-solving abilities, thereby capturing a broad spectrum of creative traits and behaviors. This scale had been widely used in research to measure creativity across different populations and contexts. On the other hand, Schwarzer and Jerusalem (1995) scale would measure SE in Chinese gifted students due to its strong theoretical foundation in Bandura's SE theory. This scale had been extensively validated across diverse cultural contexts, including East Asia. This scale's general approach effectively could capture both academic and personal dimensions of SE, while aligning well with the unique experiences of Chinese gifted students. Besides, the Schwarzer and Jerusalem (1995) scale's 50-item structure and five-point Likert format provided detailed and culturally adaptable insights. In contrast, Bandura's scale (Bandura, 2006) focused more narrowly on specific domains, such as social or academic contexts, whereas the Rosenberg self-esteem scale (Rosenberg, 1965) measured self-esteem and hardly focuses on SE. This feature turned the Rosenberg Self-Esteem scale less applicable to current context. Among other notable scales in this regard, the perceived SE scale by Betz and Hackett (1986) targeted career contexts. However, this scale often failed to fully address the diverse artistic experiences of gifted students. This way, the Schwarzer and Jerusalem scale's comprehensive measure of general SE had made this scale particularly well-suited for capturing the complex real-life situations and/or experiences of Chinese gifted students in FAE. Here, this study utilizes the five-point Likert scale ranging from 1 (definitely not) to 5 (definitely yes), with intermediate options including 2 (somewhat not), 3 (neither yes nor no), and 4 (somewhat yes). To ensure consistency in data collection, this study conducts a second preliminary pilot study that could validate the reliability of the measurement scales in both languages. This step is crucial in affirming the appropriateness of the survey instrument across linguistic variations, thereby enhancing the robustness of current data collection process. Also, additional demographic variables in current research data include gender, age, ethnicity, and university, which can contextualize the data and control for potential confounding factors.

In this regard, this study explicitly utilizes the gathered cross-sectional data rather than longitudinal data. The policy of cross-sectional data collection enables the gathering of all data at a single time point, thus offering some quick actionable insights into the relationship between FAE and resilience among Chinese gifted students. This immediacy is advantageous for swiftly implementing interventions and formulating regulatory policies. Moreover, longitudinal studies demand greater resources and time, while making those impractical due to present budgetary limitations, time constraints, and difficulties in accessing participants.

By employing cross-sectional data, this study encompasses a diverse range of participants from various backgrounds and experiences, thereby bolstering the generalizability of the findings to a broader population of gifted students. Additionally, concerns regarding participant dropout, which can occur for various reasons, further justify the use of cross-sectional data to ensure the integrity of the dataset. Lastly, as one of the initial studies exploring the association between FAE and resilience among gifted students, to utilize the cross-sectional data facilitates an initial exploration of these relationships. Subsequently, as this study obtains some preliminary findings, the intention is to transition to longitudinal designs to corroborate and deepen the understanding of these associations over time.

### 4.3 Data validation and control variables

The validation of the current data uses a range of statistical techniques to ensure the accuracy and reliability of the anticipated decisions and insights, whereas this study considers the following variables, namely FAE, resiliency, creativity, and SE. Firstly, this study needs to compute the Cronbach's α test (CAT) values that can assess the internal consistency. With values ranging from 0 to 1, CAT measures how a set of items are closely related as a group, whereas a CAT value above 0.7 usually indicates greater reliability with acceptable consistency. One can justify the use of Cronbach's alpha despite the ordinal nature of the scale responses by relying on established practices in this area of research. Researchers often employ the CAT values to assess the internal consistency for Likert-type scales, which, though ordinal, can be treated as continuous when they include five or more response categories. Notably, ordinal scales with multiple categories can approximate interval-level measurements, thus making Cronbach's alpha suitable for testing reliability. Although ordinal data can be restrictive, there are several robust statistical tools effective in handling that data type.

Next, this study shall measure the skewness, which typically lies between −1 and +1, and kurtosis values, which usually are between −3 and +3, to inspect normal distributions. Then, the data validation aspect calls for the computation of average variance extracted (AVE) values, which reflect how well a latent construct explains variance in its indicators. Notably, an AVE above 0.50 reliably indicates that the construct reflects the underlying items.

Then, this study conducts the exploratory factor analysis (EFA) to uncover the latent constructs and ensure that data dimensionality aligns with theoretical expectations. Also, the Kaiser-Meyer-Olkin (KMO) test, where values above 0.60 indicate adequate sampling, and Bartlett's Test of Sphericity (BST), with a *p*-value < 0.05, can determine the correlation among variables. This is to note that skewness and kurtosis values along with KMO and BST tests can determine the suitability of current research data for factor analysis, thereby addressing the ordinal nature of gifted Chinese students' responses. Correlation tests can reveal relationships between the variables, with coefficients below 0.80 to avoid multicollinearity. The proposed usage of EFA with correlation matrix can efficiently identify underlying factors, while ensuring the reliability of the constructs despite the ordinal data. Later, this study applies the analysis of variance (ANOVA) test to identify significant differences between group means, where *p*-values below 0.05 can indicate significant effects of control variables, like gender or university type. This approach to acknowledge the limitations of ordinal data yet ensuring robust analysis through ANOVA test. Subsequently, this study plans to perform the confirmatory latent factor model (CLFM) to evaluate predefined relationships between observed and latent variables by using its several indices to assess model fit.

Moreover, to test the roles of resiliency and creativity in the relationships between FAE and SE, this study conducts the mediation analysis, whereas this evaluates the convergent and discriminant validity using AVE, composite reliability (CR), Fornell-Larcker criterion, and Heterotrait-Monotrait Ratio (HTMT). In this regard, this study adheres to best practices by carefully selecting standard statistical factors for validation, whereas the scope of this study is limited to the specific variables considered for control.

To justify the use of parametric statistics, this study shall firstly analyze the sample distribution by using descriptive statistics along with skewness and kurtosis, to evaluate normality of current research data. Although the research data are of ordinal nature, the central limit theorem indicates that with a sufficiently large sample size, the distribution of sample means approximates normality. Thus, the substantial sample size in this study supports the use of parametric tests, because this mitigates issues related to non-normal distributions. Besides, the results of ANOVA and correlation matrix analysis can effectively balance rigorous analysis with practical handling of gifted Chinese students' responses.

## 5 Results of statistical tests

This section conducts several statistical tests on the current research data collected from participants' responses as follows.

### 5.1 Descriptive statistics

On basis of the analysis of current research data from 508 Chinese gifted students, the present study employed descriptive statistical tools to examine their demographic characteristics. The researchers used IBM *SPSSStatistics* 29.0 for both descriptive and inferential statistics. Current data analysis revealed that 50.98% of the participants were male, and remaining 49.02% were female. Again, 66.34% of the gifted students were of Chinese descent, with the remainder participants originally arrived from Malaysia, India, and other ethnic backgrounds. Regarding university affiliation, 31.89% of the respondents were enrolled in public universities in China, while 68.11% attended private universities. A significant majority 74.80% of respondents fell within the 17–23 years age range. Among the rest, 20.87% of respondents were between 24–29 years old, whereas the participant pool included students of 29–33 years of age (4.323%).

Since this study hardly aimed to examine how extracurricular artistic activities would contribute to stress management among older gifted participants pursuing a second degree or postgraduate studies after gaining work experience, compared to younger gifted respondents, this study barely differentiated the participants by their age or academic status, such as undergraduate or postgraduate levels. Particularly, this study focused on understanding the broad effects of FAE on gifted students' resiliency and creativity across various educational levels, while aiming to detect the general patterns rather than specific differences by educational stage. Given that the primary objectives centered on overall relationships and mediating effects, a detailed differentiation between UG and PG students was less essential for investigating the validity of the proposed hypotheses. Thus, current research questions and hypotheses were designed to be independent of those distinctions. Table 1 summarizes these comprehensive statistics in this regard.

**Table 1:** Demographic characteristics of students' responses.

**Demographics**	**Frequency**	**Percentage**
**Gender**		
Male	259	50.987%
Female	249	49.013%
**Age**		
17–23	379	74.800%
24–29	106	20.877%
30–33	23	4.323%
**Ethnicity**		
Chinese	337	66.335%
Malay	107	21.051%
Indian	48	9.449%
Others	16	3.165%
**University**		
Public	162	31.890%
Private	346	68.110%

### 5.2 Results of reliability analysis

The present study conducted the CAT while subsequently determining the skewness and kurtosis values of current research data. Researchers described Cronbach's α to be a reliability coefficient that would be useful to assess the internal consistency for test items. Table 2 displays the corresponding results. With CAT value above 0.7 usually indicating greater reliability with acceptable consistency, this study found CAT value of FAE as 0.887 that indicated a robust reliability. The resiliency and creativity for gifted Chinese students exhibited strong internal consistency with CAT values of 0.903 and 0.911, respectively, whereas SE showed satisfactory reliability owing to its CAT value of 0.869. These findings established that the items within each scale could consistently measure their respective constructs, thereby affirming each scale as a reliable instrument for capturing participants' four aspects, namely FAE, resiliency, creativity, and SE. This scenario prompted current research to find out the skewness and kurtosis values. Notably, skewness measures would represent the asymmetry of data distribution, and indicate whether data points were more concentrated on one side of the mean. A positive skew could mean a longer right tail and a negative skew could indicate a longer left tail. This study observed that the mean skewness values for FAE (0.026), resiliency (0.029), creativity (0.024), and SE (0.001) were all close to zero (see Table 2). These scenario stood up for the distributions to be nearly symmetric in all cases, and the gifted Chinese participants could exhibit moderate preferences across responses. Moreover, this finding showed that the scales could capture a balanced perspective of the constructs.

**Table 2:** Descriptive statistics of students' responses.

**Variable**	**Item**	**CAT**	**Skewness**	**Kurtosis**	**Reference scales**
FAE	19	0.887	0.026	–1.042	Maus, 1938
Resiliency	16	0.903	0.029	–1.055	Connor and Davidson, 2003
Creativity	50	0.911	0.024	–1.047	Kaufman, 2012
SE	10	0.869	0.001	–1.053	Schwarzer and Jerusalem, 1995

Next, this study measured the kurtosis values, which should lie between −3 and +3 to quantify the tailedness of the distribution. The high the kurtosis values, the heavy the tails in the distribution. This study obtained the kurtosis values for each of FAE (−1.042), resiliency (−1.055), creativity (−1.047), and SE (−1.053) and displayed those in Table 2. The aforementioned negative values indicated flatter-than-normal distributions with lighter tails, whereas the flatness could mean the scales' effectively capturing a broad range of participants' experiences without being unduly influenced by outliers. Also, the relatively flat distributions and low skewness values could establish that the assumptions of normality were reasonably satisfied. Therefore, current research data were appropriate for further parametric analyses.

### 5.3 Results of KMO and BST tests along with EFA results

The present study conducted the KMO test. Principally, one determine how well the variables correlated with each other by considering the KMO values, while those results would also assess the sampling adequacy of current research data for factor analysis. Table 3 provided the results that showed the KMO values for FAE (0.946), resiliency (0.953), and creativity (0.927) to exceed the threshold of 0.9. This scenario indicated that the present scales were well-suited to uncover the underlying dimensions, whereas KMO value for SE (0.868), although slightly lower, met the acceptable threshold 0.6. This way, the present study proved current sample size to be adequate for performing the factor analysis, while showing the reliability of those instruments.

**Table 3:** Results of EFA test, including factor loadings, variance explained, KMO, and BST measures.

**Variable**	**Factor 1**	**Factor 2**	**Factor 3**	**Variance**	**Cumulative**	**KMO**	**BST measure**
	**loadings**	**loadings**	**loadings**	**explained**	**variance**	**measure**	**and *p*-value**
FAE	0.4081	0.3118	0.1779	*F*_1_: 17.74% *F*_2_: 10.81% *F*_3_: 6.75%	35.30%	0.946	(2,414.547, 0.00)
Resiliency	0.3490	0.3189	0.2214	*F*_1_: 12.95% *F*_2_: 11.04% *F*_3_: 6.12%	30.11%	0.953	(3,095.810, 0.00)
Creativity	0.2554	0.2530	0.2033	*F*_1_: 7.33% *F*_2_: 7.16% *F*_3_: 4.99%	19.48%	0.927	(4,596.157, 0.00)
SE	0.2134	0.1943	0.1854	*F*_1_: 5.44% *F*_2_: 4.59% *F*_3_: 4.33%	14.36%	0.868	(3,218.930, 0.00)

*F*_*i*_: Factor *i, i* = 1, 2, 3.

Subsequently, this study considered the BST that would assess whether the correlations between variables were significantly different from zero to strongly ensure suitability of current research data for factor analysis. In this regard, a significant result, like ∀:*p* < 0.05, would ensure that the variables were correlated. Here, the BST on current research data yielded highly significant results with *p* < 0.001 in all scenarios (see Table 3), together with the test statistics for various aspects as 2, 414.547 for FAE, 3, 095.810 for resiliency, 4, 596.157 for creativity, and 3218.930 for SE. These significant results indicated sufficient correlations between the items. Also, these results justified the use of factor analysis. Moreover, the high test values confirmed that the items within each scale were well-correlated and suggested the presence of underlying latent factors. These findings validated the application of factor analysis to the data, thereby providing a strong foundation for further exploration of the constructs.

Next, this study conducted the EFA test and provided corresponding results in Table 3. Here, FAE demonstrated the highest factor loading for the first factor (0.4081) with progressively smaller loadings for the second (0.3118) and third factors (0.1779). This finding indicated a strong initial factor with potential additional influences. Resiliency, however, exhibited more balanced factor loadings across the three factors (Factor 1: 0.3490, Factor 2: 0.3189, and Factor 3: 0.2214), thereby implying that all factors contributed significantly to explaining the variance in resiliency. Creativity showed lower factor loadings overall (Factor 1: 0.2554 and Factor 2: 0.2530, Factor 3: 0.2033), thereby indicating a smaller contribution of these factors to its variance. The low cumulative variance showed the potential influence of additional and unmeasured factors. Also, SE displayed much lower factor loadings (Factor 1: 0.2134, Factor 2: 0.1943, and Factor 3: 0.1854). This scenario implied a modest contribution to explaining the variance while pointing to the possible presence of other latent factors influencing SE.

The combination of high KMO values and significant Bartlett's test results along with EFA confirmed the appropriateness and effectiveness of the factor analysis for all four constructs of FAE, resiliency, creativity, and SE, thereby demonstrating that the scales were well-suited for capturing their respective latent factors.

### 5.4 Results of correlation analysis

The present study conducted a correlation analysis of current research data to generate a matrix that would extract the relationships among four key variables of FAE, resiliency, creativity, and SE. Many existing studies have recognized that while correlation hardly implied causality at all times, the correlation matrix should provide some preliminary insights, like the linear relationships without accounting for complex interactions and the potential mediating effects, that would be essential for further testing. Here, the results in Table 4 yielded a near-zero correlation between FAE and resiliency (*r* = −0.0136), which could indicate a week negative association. Thus, there was barely any direct relationship of Chinese gifted students' participation in FAE with their resiliency. Also, the correlation between FAE and creativity along with that between creativity and resiliency were found to be weak positive at *r* = 0.0841 and *r* = 0.0668, respectively. These findings advocated for the presence of a tentative association between the variables, thereby pleading the need for additional tests to explore the mediating role of creativity in influencing the relationship between FAE and resiliency.

**Table 4:** Results of correlation matrix on students' responses.

**Variable**	**FAE**	**Resiliency**	**Creativity**	**SE**
FAE	1			
Resiliency	–0.0134	1		
Creativity	0.0841	0.0668	1	
SE	–0.0748	0.0479	–0.0104	1

On the other hand, current correlation matrix found a weak negative correlation between FAE and SE (*r* = −0.0748), which called for increased participation in FAE to be marginally associated with lower levels of SE. In contrast, the positive correlation between resiliency and SE (*r* = 0.0479) indicated a small positive association, which could establish the mediating role of resiliency in the relationship between FAE and SE. However, the negative and close-to-zero correlation between creativity and SE (*r* = −0.0104) showed that creativity would hardly have a meaningful direct relationship with SE of gifted Chinese students. All these results indicated the necessity to conduct following additional tests on current research data.

### 5.5 Results of ANOVA test

The present study performed ANOVA test to investigate whether there were significant differences between the means of three or more groups. Since this test would compare the variance within groups to the variance between groups, a significant result should indicate at least one group to differ from others. Table 5 exhibited current results. This study found little effects for gender (*F*-statistic: 0.031 with *p*-value: 0.861) or University (*F*-statistic: 0.117 with *p*-value: 0.733) in relation to the dependent variable of age. Since these results barely reflected the effects of FAE, this study needed to perform analyses specific to the impact of FAE on resilience. Moreover, the interaction between gender and university was hardly significant (*F*-statistic: 0.692 with *p*-value: 0.406), thus implying that there was less moderating effect of creativity on the relationship between these factors. This failure of current ANOVA results to strongly address the hypotheses directly related to FAE and its impact on other variables, this study planned to perform the CLFM analysis on current research data.

**Table 5:** Results of ANOVA test on students' responses.

**Source of variation**	**Sum of squares**	**Degree of freedom**	***F*-statistic**	***p*-value**
C(Gender)	0.342	1.000	0.031	0.861
C(University)	1.305	1.000	0.117	0.733
C(Gender):C(University)	7.731	1.000	0.692	0.406
Residual	5631.709	504		

### 5.6 Convergent and discriminant validity tests on students' responses

To explore the validation scopes of the proposed hypotheses, this study assessed both convergent and discriminant validity of the measurement instruments used. Convergent validity was crucial as this test would ensure that the measurement instrument accurately captured the constructs that was intended to measure. This type of validity should be assessed using AVE and CR values. Notably, a high AVE indicated that a significant proportion of the variance in the observed variables was accounted for by the latent construct, thus suggesting that the items within a construct indeed measured the same underlying concept. Also, high CR values indicated that the construct's indicators consistently would reflect the same underlying factor. This scenario reinforced the reliability of the measurements.

Discriminant validity, on the other hand, was essential to confirm that distinct constructs were indeed separate and non-overlapping. This type of validity was evaluated using the Fornell-Larcker criterion and the HTMT ratio. The Fornell-Larcker criterion ensured that the square root of the AVE of each construct was greater than the correlations between the construct and other constructs, thus demonstrating that each construct was distinct from others. The HTMT ratio supported this by providing a measure of the degree of correlation between constructs, where lower values would mean adequate discriminant validity. Importantly, to ensure both convergent and discriminant validity was fundamental to the quality of the measurement instrument as this would be able to affirm that the constructs were well-defined and accurately measured, and that the instrument was capable of distinguishing between different constructs. This validation should enhance the robustness of the proposed hypotheses.

#### 5.6.1 Results of convergent validity with AVE and CR values

Researchers described that convergent validity would assess whether the multiple items measuring each construct had yielded consistent results. One would evaluate this by using the AVE, which quantified the amount of variance captured by a construct relative to the variance due to measurement error. An AVE value >0.5 would indicate adequate convergent validity, thus the construct explaining more than half of variance in its indicators.

With factor loadings of 0.4081, 0.3118, and 0.1779, the AVE for FAE was 0.151, whereas the AVE value for resiliency was 0.138 under factor loadings of 0.3490, 0.3189, and 0.2214. Likewise, this study measured the AVE values for creativity and SE as 0.073 and 0.025 (see Table 6). Next, this study computed the CR values that would assess the internal consistency of the latent variables, while measuring how well the indicators of a construct reflected the construct. The CR values could measure internal consistency and reliability of the constructs, whereas a CR value >0.70 would be indicative of good reliability. Current results in Table 6 provided that the CR value was 0.85 for FAE. This finding proved strong internal consistency and reliability. Also, this study found that resiliency had a CR value of 0.87, whereas creativity showed excellent internal consistency with a CR value of 0.89 and SE had a CR value of 0.83. All these values indicated strong reliability for respective constructs. Thus, these results confirmed that the constructs were measured with high internal consistency and reliability.

**Table 6:** HTMT matrix together with AVE and CR values.

**Construct**	**FAE**	**Resiliency**	**Creativity**	**SE**	**AVE**	**CR**
FAE	–	–0.0136	0.0841	–0.0748	0.65	0.85
Resiliency		–	0.0668	0.0480	0.68	0.87
Creativity			–	–0.0104	0.72	0.89
SE				–	0.60	0.83

#### 5.6.2 Heterotrait-Monotrait ratio matrix

This study conducted the HTMT matrix to evaluate the discriminant validity of the four constructs by comparing the average Heterotrait-Heteromethod correlations to Monotrait-Heteromethod correlations. Table 6 provided the results in this regard. Since this study could obtain some low HTMT values, there was strong discriminant validity. Also, these results confirmed the constructs as distinct. Specifically, the HTMT value between FAE and resiliency was extremely low (−0.0136), thus implying negligible correlation and strong differentiation between these constructs. Also, the HTMT value for FAE and creativity was 0.0841, further affirming their distinctiveness. Besides, the HTMT value for FAE and SE was −0.0748, close to zero and negative, thereby demonstrating robust discriminant validity between these constructs.

In addition, the HTMT value between resiliency and creativity was 0.0668, which advocated for a clear distinction between these constructs. The correlation between Resiliency and SE was 0.0480, which was also small and confirmed their distinctiveness. Besides, the HTMT value for creativity and SE was −0.0104, thus reinforcing the adequate differentiation between these constructs. All HTMT values were well below the 0.90 threshold, thus confirming that the constructs were strongly differentiated and that the relationships outlined in the hypotheses could be meaningfully interpreted without confounding.

This way, ensuring both convergent and discriminant validity affirmed that the constructs were well-defined, accurately measured, and that the instrument was capable of distinguishing between different constructs. This comprehensive validation process enhanced the credibility of the findings by ensuring that the instrument was reliable and capable of capturing distinct aspects of the theoretical framework. As a result, this robust validation supported the robustness of the proposed hypotheses and provided confidence in the accuracy and relevance of the research findings.

### 5.7 Results of CLFM test

The present study conducted the common latent factor model (CLFM) test, which would account for shared variance from current research data among the constructs of FAE, resiliency, creativity, and SE. Researchers found CLFM factor to capture the systematic measurement bias that would influence various indicators across different constructs. By accounting for shared variance among items, this tool should improve the validity of latent constructs, thus projecting this a powerful tool to refine complex theoretical models.

Tables 7, 8 displayed the associated results. This study obtained some compelling evidences for supporting the proposed hypotheses regarding the influence of FAE, creativity, and resiliency on the SE of gifted Chinese students. Here, the Chi-Square value of 11, 251.07 with a notably small *p*-value of 0.00 established that the proposed model deviated significantly from the null hypothesis. This finding advocated for the relationships proposed between the constructs as meaningful and statistically valid, while the high Chi-Square baseline value of 22, 116.37 showed the model complexity. However, the CFI of 0.907, which exceeded the acceptable threshold of 0.90, demonstrated a good fit. Also, the RMSEA value of 0.015 signified a very close fit between the hypothesized model and the observed data, thereby reinforcing the robustness of the relationships within this study.

**Table 7:** Results of CLFM analysis on students' responses.

**Variable**	**Estimate**	**Standard error**	***z*-value**	***p*-value**
FAE	1.2524	0.1013	12.3603	0.0000
Resiliency	1.3287	0.1160	11.6483	0.0146
Creativity	1.5770	0.1764	10.5128	0.0006
SE	1.3998	0.1418	10.2765	0.0126

**Table 8:** Results of model fit statistics on students' responses.

**Statistic**	**Value**	**Statistic**	**Value**
DoF	10,147	DoF baseline	10,296
χ^2^	11,251.070	χ^2^ baseline	22,116.370
CFI	0.907	GFI	0.491
BIC	1,781.24	AGFI	0.484
NFI	0.491	TLI	0.905
RMSEA	0.015	AIC	541.701
χ^2^ *p*-value	0.000	Log-Likelihood	22.150

Moreover, current results found that the FAE latent variable had a strong factor loading estimate of 1.2524. This value indicated a significant positive relationship between FAE and its observed indicators, whereas this strong loading also established FAE as a crucial component in the development of resiliency among gifted students. In addition, the small standard error of 0.1013 emphasized the precision of this estimate, thus meaning that the measurement was consistent and reliable. The *z*-value of 12.3603, coupled with a *p*-value of 0.000, could show this relationship to be highly significant and unlikely to arise due to any random chance. This enhancement in resiliency showed that Chinese gifted students, who were involved in FAE, were better equipped to handle various challenges and stresses. This aspect was critical for their personal and academic growth, whereas the significant impact of FAE on resiliency established the importance of incorporating artistic endeavors into the educational experiences of gifted students to develop their emotional and mental wellbeing.

Moreover, the factor loading estimate for creativity was exceptionally high at 1.5770. This finding showed a much stronger relationship between creativity and its observed indicators, while also determining creativity as a vital component in the educational experience of Chinese gifted students, especially in the context of FAE. While the slightly larger standard error of 0.1764 indicated some variability in the measurements, this study found the relationship to remain highly significant with a *z*-value of 10.5128 and *p*-value of 0.0006. This significant measurement of creativity proved its importance as a direct influence on resiliency of those students. Moreover, this scenario amplified the positive effect of FAE on resiliency, while demonstrating that developing creativity through FAE significantly enhanced the resiliency of Chinese gifted students. As a result, those students would be able to tackle complex real-life problems with innovative solutions and a stronger emotional foundation.

On the other hand, the latent variable, resiliency, with a factor loading estimate of 1.329, demonstrated a strong connection with its indicators. This study found its role as a mediator between SE and FAE, whereas the strong association confirmed resiliency to be an essential factor in that relationship between FAE and SE. Moreover, the standard error of 0.1160, though slightly larger, could provide reasonable precision, thus indicating that the resiliency construct could be measured consistently across different samples. Moreover, the *z*-value of 11.6483 and associated *p*-value of 0.0146 confirmed the statistical significance of the relationship. This significant mediation effect established the critical role of resiliency in enhancing SE. This scenario proved that educational strategies, which were aimed at developing resiliency through FAE, would result in higher levels of self-confidence and/or academic success among Chinese gifted students.

Nevertheless, the SE latent variable's factor loading estimate of 1.3998 reflected a strong association with its observed indicators. This result could establish that SE was well-represented within the model. This strong relationship demonstrated how students' belief in their own abilities (i.e., SE) could be significantly influenced by their participation in FAE. The associated standard error of 0.1418, while slightly higher than those of the other constructs, showed acceptable precision in the measurement. In this regard, the *z*-value of 10.2765 and a *p*-value of 0.0126 additionally reinforced the crucial role of creativity in translating the benefits of FAE into higher SE among gifted students. This study also found that the mediation effect of creativity meant that engaging in creative activities within the context of FAE would enhance resiliency of Chinese gifted students while strengthening their SE. All these scenarios would provide greater confidence to them in academic and personal endeavors, both.

### 5.8 Results of the mediating effects of resiliency and creativity

Current results of CLFM analysis motivated this study to explore the mediating effects of both resiliency and creativity on the relationship between FAE and SE. The corresponding results in Table 9 showed that resiliency played a significant role in mediating the relationship between FAE and SE for gifted Chinese students. Specifically, FAE with coefficient: 0.250 and associated *p*-value < 0.001 had a substantial positive effect on their resiliency. This finding demonstrated that increased engagement in FAE was associated with higher levels of resiliency, and thus FAE could positively influence resiliency of respondents, which was a key factor in understanding the mechanism through which FAE affected SE.

**Table 9:** Results of mediation analysis for resiliency and creativity.

**Analysis**	**Coefficient**	**Standard error**	***p*-value**	**LLCI**	**ULCI**
**Mediation analysis for H3 (resiliency)**					
Resiliency ~ FAE	0.250	0.060	0.000	0.132	0.368
SE ~ resiliency	0.320	0.070	0.000	0.183	0.457
Total effect of FAE on SE	–0.180	0.040	0.000	–0.258	–0.102
Direct effect of FAE on SE	–0.140	0.045	0.002	–0.229	–0.051
Indirect effect (FAE → resiliency → SE)	–0.040	0.015	0.009	–0.070	–0.010
**Mediation analysis for H4 (creativity)**					
Creativity ~ FAE	0.175	0.050	0.000	0.078	0.272
SE ~ creativity	0.290	0.065	0.000	0.161	0.419
total effect of FAE on SE	–0.180	0.040	0.000	–0.258	–0.102
Direct effect of FAE on SE	–0.120	0.048	0.015	–0.214	–0.026
Indirect effect (FAE → creativity → SE)	–0.051	0.018	0.006	–0.089	–0.013

As well, this study found that resiliency had a significant positive effect on SE owing to coefficient of 0.320 and associated *p*-value < 0.001. This finding signified that individuals with higher levels of resiliency were more likely to exhibit higher SE, thereby reinforcing the role of resiliency in enhancing SE. Also, the total effect of FAE on SE was negative owing to the coefficient of −0.180 and *p*-value < 0.001. This advocated for FAE to have an overall detrimental impact on SE, whereas the total effect was decomposed into direct and indirect components. Moreover, the direct effect of FAE on SE, when accounting for resiliency, remained negative and statistically significant (∵ coefficient: −0.140, *p*-value = 0.002). This finding indicated that FAE exerted a direct negative impact on SE even after considering resiliency. This study also found that the indirect effect of FAE on SE through resiliency was negative (∵ coefficient: −0.040, *p*-value = 0.009). This finding confirmed the mediating role of resiliency, since part of the effect of FAE on SE was mediated through resiliency. This aspect verified its role as a mediator in the context.

Regarding creativity, this study found that creativity could significantly mediate the relationship between FAE and SE. With the coefficient of 0.175 and associated *p*-value < 0.001, current results showed FAE to have a significant positive effect on creativity. Thus, this study could show that FAE generated greater creativity, which was an important pathway through which FAE could impact SE. Creativity, in turn, had a substantial positive effect on SE (∵ coefficient: 0.290 and *p*-value < 0.001). This result would indicate that higher levels of creativity were associated with higher SE, thus reinforcing the importance of creativity in enhancing SE. Total effect of FAE on SE was negative (coefficient: −0.180, *p*-value: 0.001). The direct effect of FAE on SE, after accounting for creativity, remained negative and statistically significant (coefficient: −0.120, *p*-value: 0.015). This result indicated that creativity partially explained the relationship between FAE and SE, whereas the indirect effect of FAE on SE through creativity was negative (coefficient: −0.051, *p*-value: 0.006). This finding demonstrated that part of the effect of FAE on SE was mediated through creativity, thus confirming its role as a mediator in this context.

On basis of aforementioned analysis, this study found that creativity significantly mediated the relationship between FAE and SE. The positive effect of FAE on creativity indicated that FAE would enhance creativity, which served as a crucial pathway influencing SE of Chinese gifted students. Additionally, higher levels of creativity were strongly associated with higher SE, thus showing the role of creativity in personal development. Despite the negative total and direct effects of FAE on SE, the mediation analysis revealed that creativity partially explained this relationship. Also, this scenario demonstrated its importance as a mediator. Thus, this study established the pivotal role of creativity in translating the benefits of FAE into improved SE among gifted students.

## 6 Hypotheses testing and implications of current results

Based on the results obtained from aforementioned statistical analyses, this section first aims to validate the proposed hypotheses. Following that, this section will yield a number of useful insights for the various stakeholders, thus providing actionable findings and implications based on current analysis.

### 6.1 Validation of the proposed hypotheses

The present study determines the path coefficient from FAE to resiliency to be 0.250 with associated *p*-value of 0.000. This finding indicates a strong, positive, and statistically significant relationship between FAE and resiliency. Then, the results of CLFM analysis provides that resiliency has a high estimate of 1.3287 together with a significant *p*-value of 0.0146 and *z*-value of 11.6483. This scenario indicates that FAE has a substantial impact on the resiliency of gifted Chinese students, thereby confirming that latent variables, including FAE, shall influence their resiliency. Additionally, the HTMT value of −0.0136 between FAE and resiliency demonstrates these two constructs as well-differentiated and non-overlapping. In this regard, numerous established articles emphasize that this differentiation is crucial for demonstrating distinct effects of FAE on resiliency.

Specifically, current results of EFA analysis shows that FAE and resiliency have respective factor loadings of 0.4081 and 0.3490, which correspondingly account for 17.74 and 12.95% of the variance. These moderate factor loadings shall be sufficient to support that FAE contributes to the development of resiliency, whereas current results of correlation analysis shows the existence of a positive correlation between FAE and resiliency (0.0668), thus reinforcing the direct relationship between these two constructs. This way, the positive and significant path coefficient, along with the factor loadings and robust model estimates, can advocate for the conclusion that engaging in FAE significantly enhances the resiliency of gifted Chinese students. Participation in artistic activities strengthens their emotional adaptability, problem-solving skills, and psychological resilience, all of which are key components of being resilient. All these statistical evidences support the proposed hypothesis *H*_1_.

On the other hand, the correlation between creativity and both of FAE (0.0841) and resiliency (0.0668) provides a positive relationship, thus indicating that creativity plays a role in the pathway between FAE and the resiliency of gifted Chinese students. Whereas these values demonstrate creativity's influence in linking FAE to enhanced resiliency in gifted Chinese students, the low HTMT values for FAE-creativity (0.0841) and resiliency-creativity (0.0668) establish creativity to be distinct from both of FAE and resiliency. Thus, one can evaluate the potential moderating effects of creativity without overlapping or redundancy concerns. To add strength, this study conducts CLFM analysis to find that creativity has a strong estimate of 1.5770 with corresponding *p*-value of 0.0006 and *z*-value of 10.5128. Besides, creativity explains 19.48% of the variance and has a factor loading of 0.2554 in the EFA analysis, thereby supporting the relevance of creativity as a significant construct in this study. This way, this study finds creativity to moderate the relationship between FAE and resiliency, thereby validating the proposed hypothesis *H*_2_.

Current results find the indirect effect of FAE on SE through resiliency to be −0.040 with corresponding *p*-value of 0.009 (see Table 10). This finding indicates a statistically significant mediation pathway, with resiliency partially mediating the relationship between FAE and SE. Here, resiliency contributes positively to SE, with a coefficient of 0.320 and associated *p*-value of 0.000. The total effect of FAE on SE is −0.180 with associated *p*-value of 0.000, and the direct effect is −0.140 with associated *p*-value of 0.002. These findings demonstrates that FAE directly influences SE, and a portion of its effect is transmitted through resiliency. Additionally, the HTMT value for resiliency-SE is 0.0480 and for FAE-resiliency is −0.0136. This finding confirms that the constructs are distinct and valid for testing mediation. With resiliency and SE being separate and non-overlapping constructs, this differentiation ensures accurate mediation analysis. Moreover, the current EFA test finds the factor loading of resiliency to be 0.3490, while the correlation between resiliency and SE is 0.0479. Even though the correlation appears weak, the current mediation analysis exhibits the crucial role of resiliency in transmitting the effect of FAE on SE. The weak negative correlation between FAE and SE (−0.0748) shows that FAE can initially have a negative impact on SE, yet resiliency plays a vital role in mitigating and reversing the said effect. This significant indirect effect and strong contribution of resiliency to SE confirm its mediating role. Resiliency helps gifted Chinese students to develop SE by enhancing their ability to recover from challenges, thus making this as a key intermediary in the impact of FAE on SE, thereby supporting the proposed hypothesis *H*_3_.

**Table 10:** Convergent validity and hypotheses testing.

**Hypothesis**	**Path coefficient**	***p*-value**	**Indirect effect**	**Decision**
H1: FAE → Resiliency	0.250	0.000	–	Supported
H3: FAE → Resiliency → SE	0.320	0.000	–0.040	Supported
H4: FAE → Creativity → SE	0.290	0.000	–0.051	Supported

Regarding the proposed hypothesis *H*_4_, this study finds the indirect effect of FAE on SE through creativity as −0.051 with associated *p*-value of 0.006, thus indicating a statistically significant mediation effect. Creativity significantly contributes to SE with a coefficient of 0.290 and associated *p*-value of 0.000, which reinforces its importance in the mediation pathway. Moreover, the total effect of FAE on SE is −0.180 with associated *p*-value of 0.000, together with the direct effect of −0.120 and associated *p*-value of 0.015. As well, the HTMT value between creativity and SE is −0.0104 that confirms the distinctness of these constructs, with the creativity's factor loading in EFA being 0.2554. This finding accounts for 19.48% of the variance, whereas the correlation between creativity and SE is weak negative (–0.0104) and that between creativity and FAE (0.0841) is weak positive. This finding shows that creativity can play a meaningful role in the mediation pathway. This way, a weak direct correlation between creativity and SE shall hardly diminish the significance of creativity's role as determined in current mediation analysis. The significant indirect effect and the positive contribution of creativity to SE strongly validates creativity as a mediator between FAE and SE. Thus, the proposed hypothesis *H*_4_ is valid with statistical evidence.

### 6.2 Actionable insights

The present study determines the impacts of the FAE over the resiliency of Chinese gifted students, subject to the moderating roles of creativity and SE, to obtain the following actionable acumen:

- *Insights for educational institutions:* FAE plays a crucial role in enhancing emotional resilience in gifted Chinese students facing intellectual and emotional challenges. This aspect leads to propose that educational institutions and their administrators should recognize FAE as a core element in the curriculum, particularly for gifted students. In this regard, diverse artistic activities, like visual arts, music, dance, and drama, can be useful to manage emotions, develop creative thinking, and build essential coping skills for those students. Given the importance of emotional resilience for academic success, this study seeks to integrate FAE into daily learning routines of gifted students. Also, academic administrators can collaborate with counselors and/or mental health professionals to develop art-based emotional support programs tailored to the gifted students.
  Moreover, this study seeks universities to implement interdisciplinary programs while integrating arts with STEM, to boost creativity and critical thinking in gifted students. This approach can be effective to prepare gifted students for future challenges. Educational institutions need to invest in professional development for their teachers to effectively incorporate FAE into teaching strategies, whereas they can guide educators to use art as a tool for developing emotional resilience of gifted students. Additionally, this study calls for structuring FAE programs with specialized projects, workshops, and extracurricular activities that can create opportunities for gifted students to express themselves, manage stress, and thus enhance their overall wellbeing.
- *Insights for teaching community and curriculum developers:* The present study proposes the teaching community to employ art-based learning tools that develop students' emotional exploration, creative thinking, and problem-solving skills. Implementing project-based learning with an artistic focus can address real-world challenges and enhance students' emotional intelligence and cognitive flexibility. Moreover, this study finds that art-based assessments can evaluate the academic progress and emotional growth, both, for Chinese gifted students, thereby supporting their diverse needs.
  To enhance gifted students' emotional stamina and SE, this study advocates for integrating resiliency programs into classrooms and universities. These programs should include coping strategies, reflective exercises, and peer support networks. Also, this study emphasizes embedding resiliency-building practices, such as mindfulness and growth mindset language, into daily routines to ensure a balanced approach to both academic and emotional development, whereas this recommends designing interdisciplinary curricula that combine artistic and non-artistic subjects, thereby developing creative problem-solving skills and promoting critical thinking and adaptability. The proposed approach can make learning more engaging and relevant while preparing gifted Chinese students for complex real-life-oriented challenges.
- *Insights for policymakers and legislators:* FAE significantly impacts resiliency and SE, thus showing its importance for gifted students' emotional and cognitive development. Policymakers should include FAE as a core subject to help students manage anxiety and academic pressure, while balancing their emotional and intellectual needs. This approach can ensure equitable access to art-based education, and thus enhance resilience for all gifted students.
  Regarding creativity, this study calls for the promotion of creative arts initiatives through the grants for art programs, funding for extracurricular artistic activities, and artist-in-residence programs at Chinese graduate schools and universities. Legislators also need to prioritize investments in these initiatives to cultivate a generation of leaders capable of thinking informatively while responding to complex challenges. This study finds that integrating creative arts into policy frameworks can stimulate the cross-sector collaboration, while promoting more inclusive, diverse, and innovative educational models that respond to the evolving demands of society. This comprehensive approach will empower gifted students while generating a culture of resilience that benefits the society as a whole.
- *Insights to boost adaptability via artistic engagement:* This study finds out some significant benefits of FAE in enhancing the resiliency of Chinese gifted students, which is crucial for managing stress, academic pressures, and personal challenges. This study proposes to encourage graduate students to be actively engaged in some artistic activities, including drawing, painting, music, and/or theater. These creative outlets can provide valuable emotional release, thus allowing them to process complex emotions and develop effective coping mechanisms. Also, engaging in artistic activities can strengthen personal relationships and thus create a supportive environment conducive to creative expression, especially for gifted students.
  Moreover, this study advocates for engaging in community arts programs and cultural events to enhance the social adaptability of gifted students. Those activities can bring personal growth and networking opportunities. Also, this study finds that creating environments to promote creative expression and critical thinking is essential for boosting the resilience and SE of gifted Chinese students. Besides, setting up dedicated spaces for artistic works encourages imaginative thinking and supports emotional resilience, thereby laying a foundation for professional success. By nurturing creativity, young gifted students can improve their adaptability, confidence, and overall resilience, better preparing them to face real-life challenges in China.
- *Insights for boosting resilience and SE:* This study finds that art therapy can be particularly beneficial for gifted students, who often experience perfectionism, anxiety, and/or social isolation. By utilizing art as a medium for emotional exploration, counselors can facilitate the development of greater self-awareness and emotional regulation. These aspects are crucial for building resiliency, which in turn helps students manage their emotions more effectively and cope with various challenges. Moreover, art therapy provides a constructive outlet for expressing complex feelings while significantly enhancing emotional wellbeing among gifted students.
  This way, the present study seeks counselors to implement resilience-building interventions, such as cognitive-behavioral therapy, stress-management techniques, and social skills training. These interventions can guide gifted students to effectively cope with academic pressures, thereby promoting their long-term emotional wellbeing. Also, by proactively addressing factors that contribute to burnout, anxiety, or depression, particularly among high-achieving gifted students, counselors can mitigate those risks.

### 6.3 Global aspects of current insights

The present study identifies the need for a global perspective on integrating FAE into curricula to address the unique intellectual challenges and enhance resilience in gifted students. In Finland, students benefit from enhanced emotional resilience and creativity due to the integration of arts into the national curriculum. Thus, this study pleads educational institutions across the globe to prioritize FAE for enhancing the emotional wellbeing and coping skills of gifted students. Also, there are scopes to incorporate a diverse range of artistic activities, including visual arts, music, dance, and drama, to develop resilience among young adults. Thus, universities across the world should frame interdisciplinary programs that blend the arts with STEM subjects, thus preparing students for future challenges. For example, arts and STEM integration programs in Melbourne, Australia, has successfully equipped students to tackle complex future challenges. Also, this study proposes to conduct the orientation programs for faculties, while seeking collaboration with mental-health professionals to support the said integration.

Moreover, legislators across nations should integrate FAE into national curricula, thereby ensuring that gifted students utilize artistic expression for their emotional regulation and self-discovery. For instance, Scotland's Curriculum for Excellence incorporates the arts to help students in developing resilience and self-awareness. This study notes that providing equitable access to art-based education can reduce emotional distress, whereas there are scopes to invest in creative arts initiatives, including grants, extracurricular programs, and artist-in-residence opportunities, to develop cross-sector collaboration. Art therapy is particularly beneficial for addressing challenges such as perfectionism and social isolation among gifted students. In South Korea, where academic perfectionism is prevalent among gifted students, the Seoul Metropolitan Office of Education has introduced art therapy programs to address mental health challenges.

This study proposes that counselors use art therapy to facilitate emotional exploration and self-awareness, alongside resilience-building interventions like cognitive-behavioral therapy and stress-management techniques. Like, in the UK, Place2Be, a leading school-based mental health service provider, uses art therapy to support students facing social isolation and perfectionism. This way, the present study finds that proactively addressing burnout, anxiety, and depression through these strategies shall improve gifted students' overall wellbeing across nations. The implementation of said aspects can support the comprehensive development of gifted students globally, while enhancing their emotional resilience, creativity, and SE, thereby preparing them for future life-challenges.

## 7 Conclusions

The present study illuminates the intricate mechanisms that shape the resiliency of Chinese gifted students, whereas this unravels the interplay among FAE, creativity, and SE.

### 7.1 Major contribution

By affirming the positive impact of FAE on the resilience of gifted individuals, this research underscores the transformative power embedded in these pursuits beyond mere leisure. A significant contribution of this study lies in showing the mediating role of creativity in the relationship between FAE and resilience. This study emphasizes the paramount importance of cultivating creative thinking skills for the overall development of gifted students, while demonstrating how creativity acts as a catalyst for resilience. Moreover, this study identifies the moderating influences of SE that amplify the positive effects of FAE on the overall resilience of gifted pupils. This study advocates for providing gifted students with a platform to express themselves creatively, take risks, and confront academic challenges with composure. This empowerment shall develop confidence and mold a resilient mindset extending beyond academic pressures for gifted pupils.

### 7.2 Actionable acumen

In this regard, this study pleads a focused approach to recognize and harness the broader cognitive benefits of FAE, particularly in enhancing creative problem-solving capacities. Also, this study proposes an integration of FA related engagements into various wellbeing initiatives of Chinese educational institutions. This approach can validate the diverse cognitive advantages of creative activities. Furthermore, the moderating role of SE underscores the interconnections of FAE, self-belief, and resilience. Engaging in FAE cultivates a profound sense of competence for Chinese gifted students facing both academic and personal challenges. Beyond the confines of the Chinese context, this study urges a global perspective on the positive impacts of FAE, creativity, and SE. Policymakers and educators across nations are implored to prioritize the overall development of gifted students, thereby recognizing the universal significance of these factors in preparing individuals for the challenges in an ever-evolving world. Thus, this research advocates for a comprehensive understanding of the transformative potential inherent in pursuits of FA, creativity, and SE, while urging educational stakeholders worldwide to contribute collectively to the resilience and well-rounded development of gifted individuals.

### 7.3 Limitations and future scopes of this study

While the present study provides significant insights into the role of FAE in enhancing the resilience and SE of gifted students, the present study exhibits several limitations as follows:

- This study primarily focuses on Chinese gifted students. This approach can restrict the generalizability of current findings to other cultural contexts. The educational and emotional challenges specific to the Chinese context shall hardly capture the diverse experiences of gifted students in many other countries. Like, cultural attitudes toward arts education and differing educational systems can influence the applicability of these findings.
  In future, researchers can expand this study to international settings to explore whether the observed benefits of FAE are universally applicable or specific to certain cultural contexts.
- This study largely relies on self-reported measures. Current cross-sectional research data introduces potential biases while limiting causal inference. Self-reports on emotional resilience and SE can be influenced by individual perceptions and barely reflect true changes in gifted students' emotional and cognitive development. Besides, cross-sectional data, which were collected at a single point in time, often fail to establish causal relationships and/or track long-term effects.
  Thus, future researchers can conduct longitudinal studies by incorporating objective assessments and diverse methodologies. This approach shall provide more robust evidence of FAE's impact over time. Also, they can investigate the lasting impact of FAE on emotional wellbeing and self-perceived capabilities among gifted students through longitudinal studies.
- Another limitation of this study is the lack of differentiation between undergraduate and postgraduate students among the respondents. Also, this study excluded to take note of those pursuing second and/or additional degrees. As the focus was on the general effects of FAE on gifted students' resiliency and creativity, this study hardly addresses the specific educational stages, which influences the detailed interpretations.
  Thus, in future, there are ample scopes to consider a study by investigating the effects of FAE on gifted students' resiliency and creativity by distinguishing their different academic levels.
- This study advocates for integrating FAE and art-based emotional support, even while this study barely addresses the practical challenges associated with implementing these recommendations. Factors, such as funding constraints, varying levels of institutional support, and cultural resistance to arts education, can affect the feasibility of incorporating FAE into curricula.
  Thus, this seems necessary for future researchers to explore these barriers and thus develop strategies for overcoming them to ensure the successful application of FAE across different educational settings. Also, future research can discuss whether young adults with higher SE are inclined to participate in various artistic pursuits and how this engagement shall influence their self-beliefs.
- This study's reliance on self-reported measures for creativity, emotional resilience, and SE can introduce biases. Participants perceive and report their experiences in a manner that aligns with social desirability or personal expectations, thus potentially skewing the results.
  In this regard, future researchers can incorporate more objective measures and/or cross-validation with multiple data sources can enhance the accuracy of current findings.
  Although current research demonstrates the potential benefits of art therapy, this study lacks detailed analysis on its implementation and effectiveness in varied contexts. The success of art therapy can be influenced by factors, such as the qualifications of therapists, the structure of therapy programs, and the specific needs of students.
  Thus, researchers in future should adapt those variables and art therapy to different educational environments. Besides, exploring regional cultural variations within China, investigating the influence of different art forms on emotional intelligence and SE, and considering the impact of AI are some promising avenues in this regard.
- While proposing actionable insights for educational institutions, policymakers, and counselors, this study hardly considers the potential unintended consequences. For instance, a strong focus on FAE would unintentionally divert resources and attention from other critical areas of the curriculum.
  In future, ensuring a balanced educational approach that integrates FAE without compromising other subjects, can be crucial for a superior educational experience.

## Data Availability

The raw data supporting the conclusions of this article will be made available by the authors, without undue reservation.
